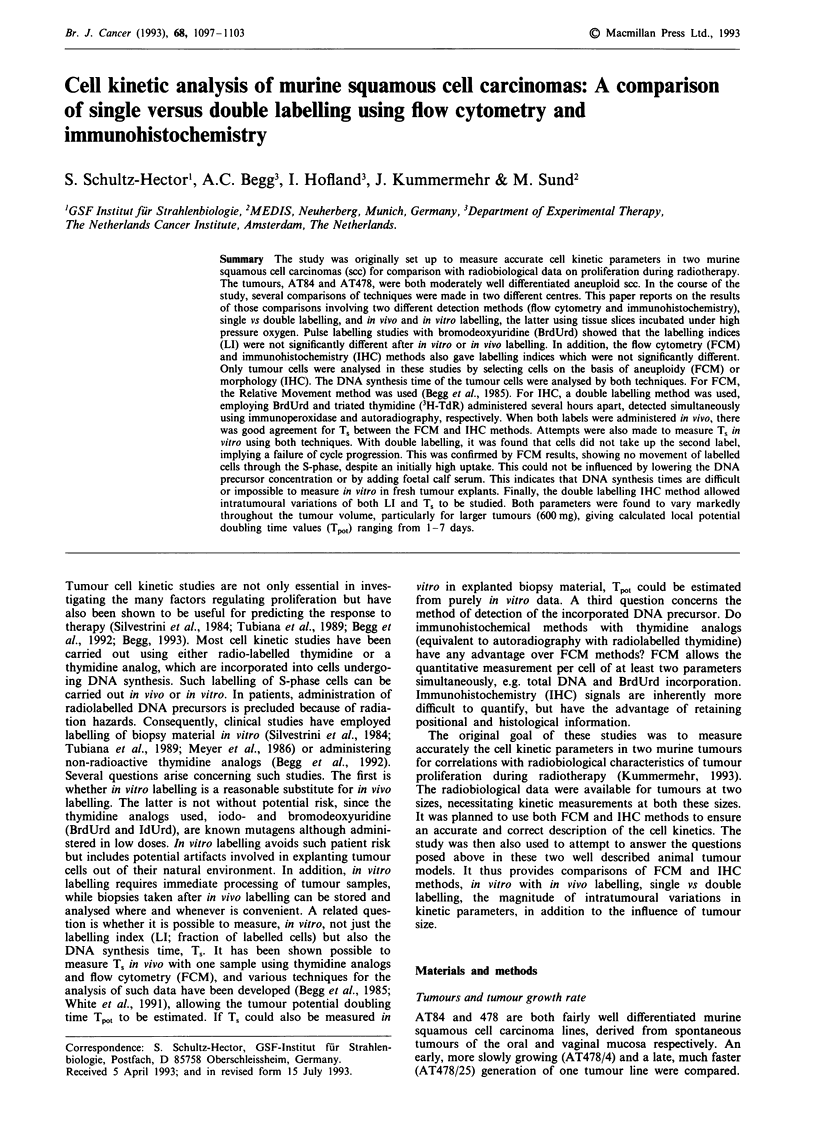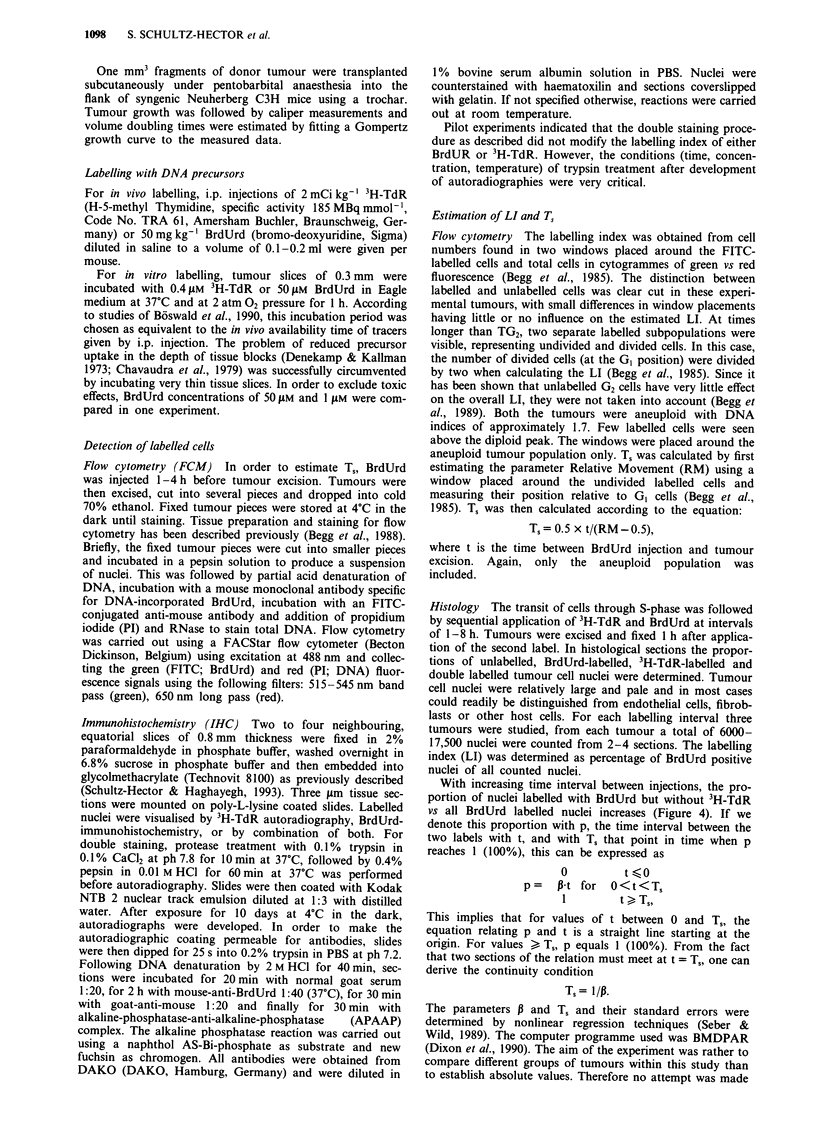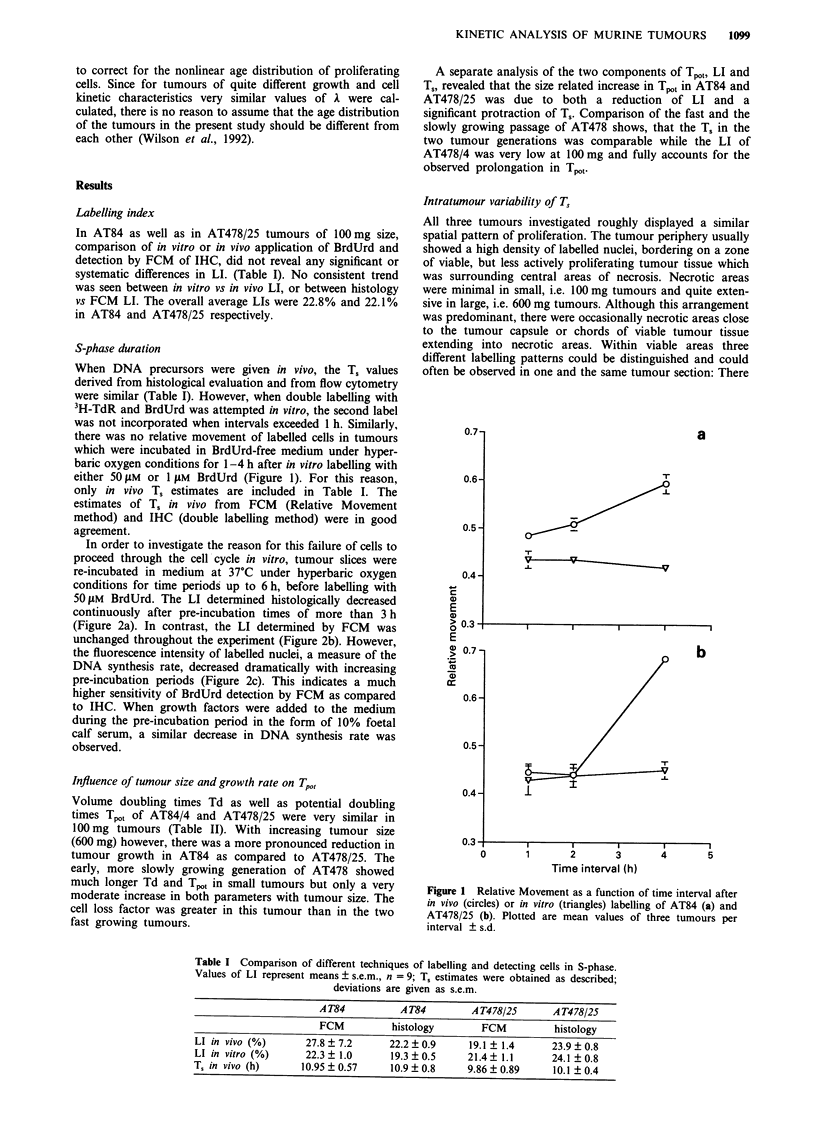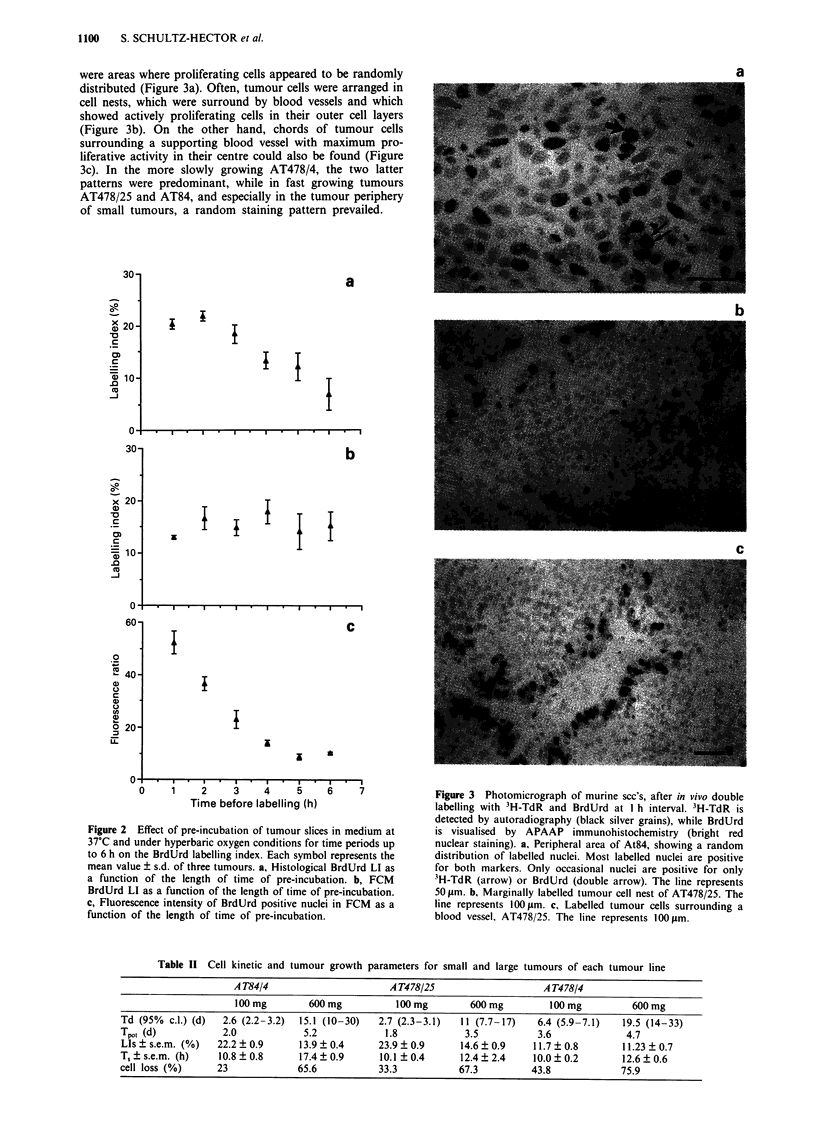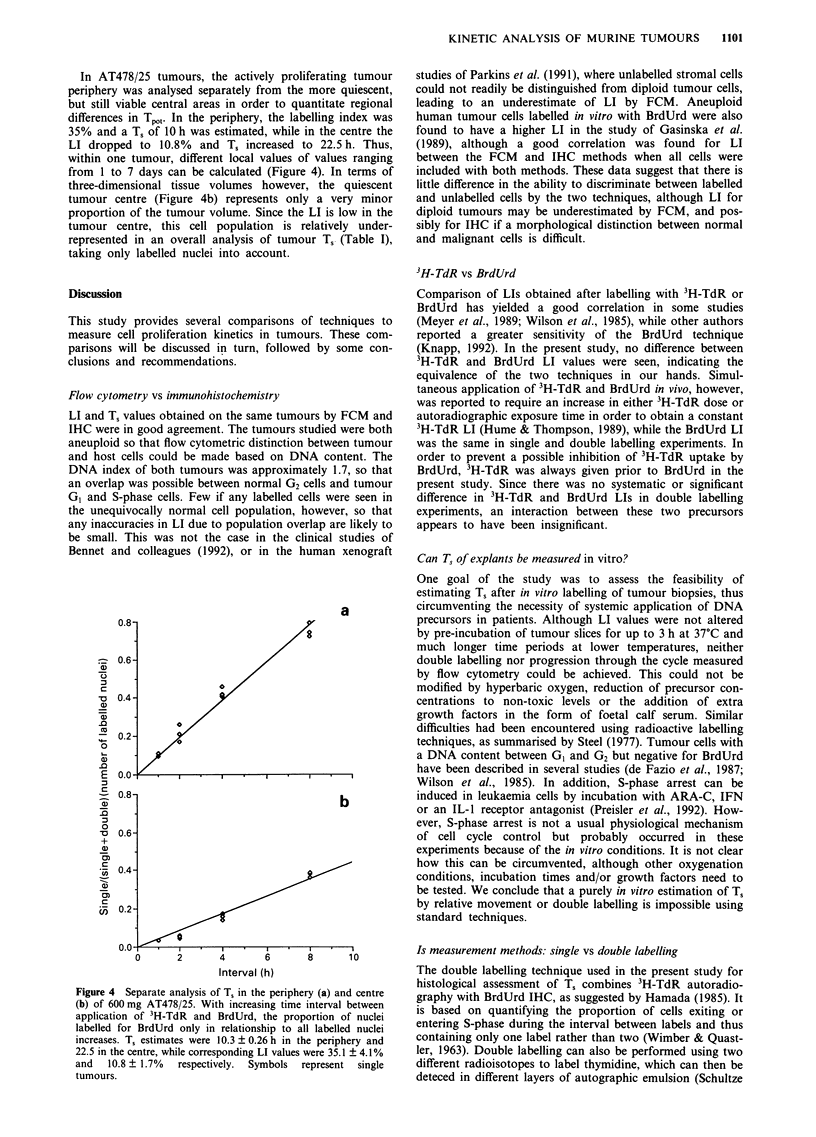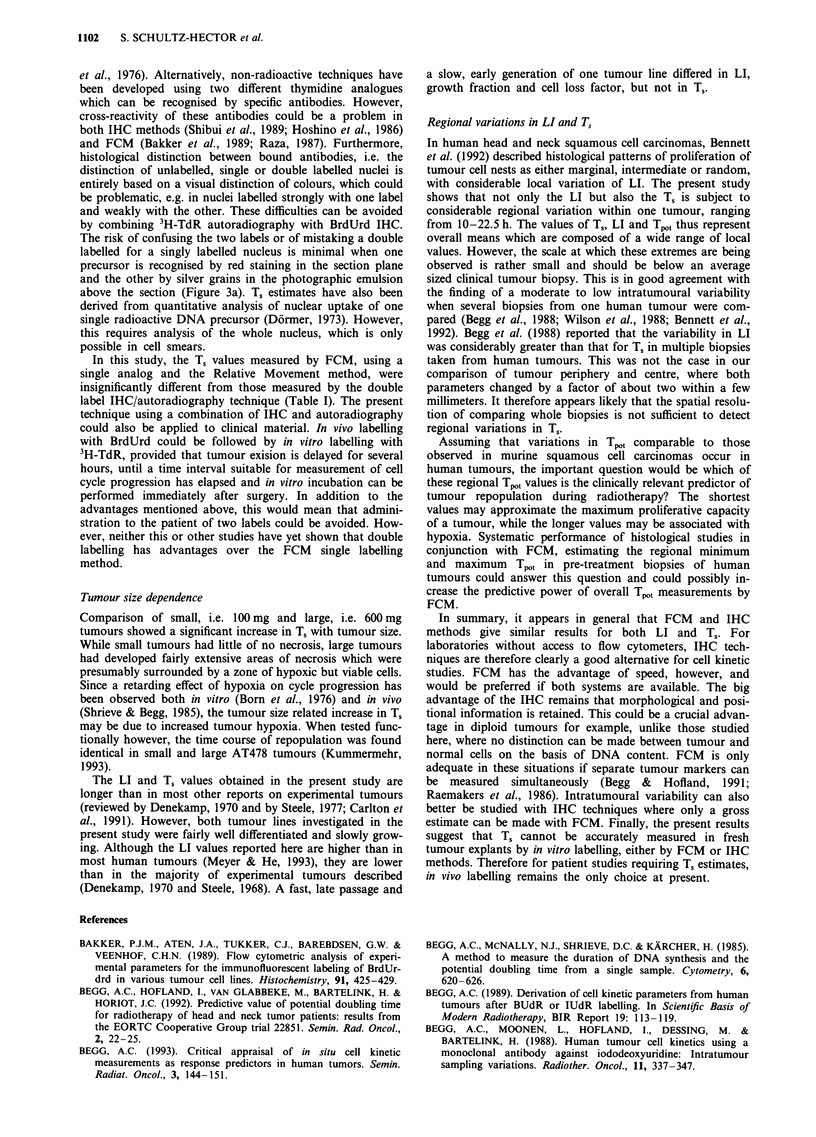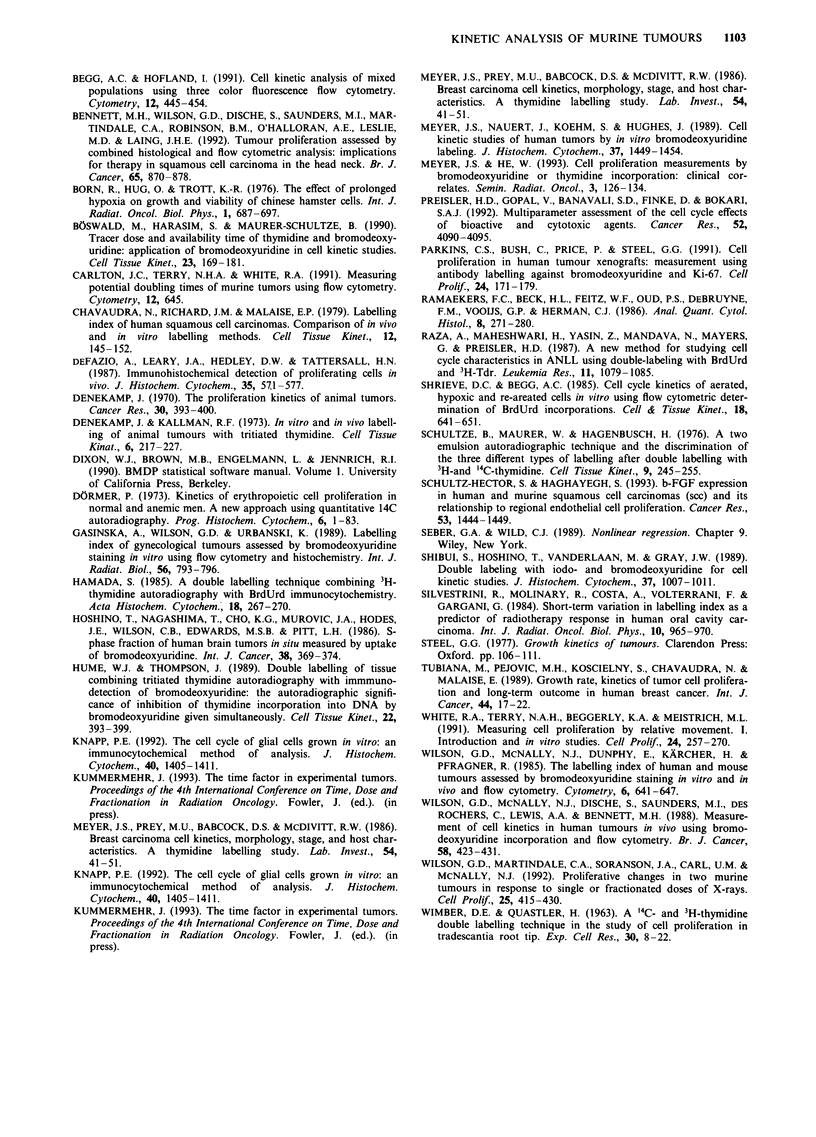# Cell kinetic analysis of murine squamous cell carcinomas: a comparison of single versus double labelling using flow cytometry and immunohistochemistry.

**DOI:** 10.1038/bjc.1993.487

**Published:** 1993-12

**Authors:** S. Schultz-Hector, A. C. Begg, I. Hofland, J. Kummermehr, M. Sund

**Affiliations:** GSF Institut für Strahlenbiologie, Neuherberg, Munich, Germany.

## Abstract

**Images:**


Br. J. Cancer (1993), 68, 1097–1103 © Macmillan Press Ltd., 1993

### Cell kinetic analysis of murine squamous cell carcinomas: A comparison of single versus double labelling using flow cytometry and immunohistochemistry

S. Schultz-Hector[1], A.C. Begg[3], I. Hofland[3], J. Kummermehr & M. Sund[2]

[1]GSF Institut für Strahlenbiologie, [2]MEDIS, Neuherberg, Munich, Germany, [3]Department of Experimental Therapy, The Netherlands Cancer Institute, Amsterdam, The Netherlands.

**Summary** The study was originally set up to measure accurate cell kinetic parameters in two murine squamous cell carcinomas (scc) for comparison with radiobiological data on proliferation during radiotherapy. The tumours, AT84 and AT478, were both moderately well differentiated aneuploid scc. In the course of the study, several comparisons of techniques were made in two different centres. This paper reports on the results of those comparisons involving two different detection methods (flow cytometry and immunohistochemistry), single *vs* double labelling, and *in vivo* and *in vitro* labelling, the latter using tissue slices incubated under high pressure oxygen. Pulse labelling studies with bromodeoxyuridine (BrdUrd) showed that the labelling indices (LI) were not significantly different after *in vitro* or *in vivo* labelling. In addition, the flow cytometry (FCM) and immunohistochemistry (IHC) methods also gave labelling indices which were not significantly different. Only tumour cells were analysed in these studies by selecting cells on the basis of aneuploidy (FCM) or morphology (IHC). The DNA synthesis time of the tumour cells were analysed by both techniques. For FCM, the Relative Movement method was used (Begg *et al.*, 1985). For IHC, a double labelling method was used, employing BrdUrd and triated thymidine ($^3$H-TdR) administered several hours apart, detected simultaneously using immunoperoxidase and autoradiography, respectively. When both labels were administered *in vivo*, there was good agreement for $T_s$ between the FCM and IHC methods. Attempts were also made to measure $T_s$ *in vitro* using both techniques. With double labelling, it was found that cells did not take up the second label, implying a failure of cycle progression. This was confirmed by FCM results, showing no movement of labelled cells through the S-phase, despite an initially high uptake. This could not be influenced by lowering the DNA precursor concentration or by adding foetal calf serum. This indicates that DNA synthesis times are difficult or impossible to measure *in vitro* in fresh tumour explants. Finally, the double labelling IHC method allowed intratumoural variations of both LI and $T_s$ to be studied. Both parameters were found to vary markedly throughout the tumour volume, particularly for larger tumours (600 mg), giving calculated local potential doubling time values ($T_{pot}$) ranging from 1–7 days.

Tumour cell kinetic studies are not only essential in investigating the many factors regulating proliferation but have also been shown to be useful for predicting the response to therapy (Silvestrini *et al.*, 1984; Tubiana *et al.*, 1989; Begg *et al.*, 1992; Begg, 1993). Most cell kinetic studies have been carried out using either radio-labelled thymidine or a thymidine analog, which are incorporated into cells undergoing DNA synthesis. Such labelling of S-phase cells can be carried out *in vivo* or *in vitro*. In patients, administration of radiolabelled DNA precursors is precluded because of radiation hazards. Consequently, clinical studies have employed labelling of biopsy material *in vitro* (Silvestrini *et al.*, 1984; Tubiana *et al.*, 1989; Meyer *et al.*, 1986) or administering non-radioactive thymidine analogs (Begg *et al.*, 1992). Several questions arise concerning such studies. The first is whether *in vitro* labelling is a reasonable substitute for *in vivo* labelling. The latter is not without potential risk, since the thymidine analogs used, iodo- and bromodeoxyuridine (BrdUrd and IdUrd), are known mutagens although administered in low doses. *In vitro* labelling avoids such patient risk but includes potential artifacts involved in explanting tumour cells out of their natural environment. In addition, *in vitro* labelling requires immediate processing of tumour samples, while biopsies taken after *in vivo* labelling can be stored and analysed where and whenever is convenient. A related question is whether it is possible to measure, *in vitro*, not just the labelling index (LI; fraction of labelled cells) but also the DNA synthesis time, $T_s$. It has been shown possible to measure $T_s$ *in vivo* with one sample using thymidine analogs and flow cytometry (FCM), and various techniques for the analysis of such data have been developed (Begg *et al.*, 1985; White *et al.*, 1991), allowing the tumour potential doubling time $T_{pot}$ to be estimated. If $T_s$ could also be measured *in vitro* in explanted biopsy material, $T_{pot}$ could be estimated from purely *in vitro* data. A third question concerns the method of detection of the incorporated DNA precursor. Do immunohistochemical methods with thymidine analogs (equivalent to autoradiography with radiolabelled thymidine) have any advantage over FCM methods? FCM allows the quantitative measurement per cell of at least two parameters simultaneously, e.g. total DNA and BrdUrd incorporation. Immunohistochemistry (IHC) signals are inherently more difficult to quantify, but have the advantage of retaining positional and histological information.

The original goal of these studies was to measure accurately the cell kinetic parameters in two murine tumours for correlations with radiobiological characteristics of tumour proliferation during radiotherapy (Kummermehr, 1993). The radiobiological data were available for tumours at two sizes, necessitating kinetic measurements at both these sizes. It was planned to use both FCM and IHC methods to ensure an accurate and correct description of the cell kinetics. The study was then also used to attempt to answer the questions posed above in these two well described animal tumour models. It thus provides comparisons of FCM and IHC methods, *in vitro* with *in vivo* labelling, single *vs* double labelling, the magnitude of intratumoural variations in kinetic parameters, in addition to the influence of tumour size.

Correspondence: S. Schultz-Hector, GSF-Institut für Strahlenbiologie, Postfach, D 85758 Oberschleissheim, Germany.
Received 5 April 1993; and in revised form 15 July 1993.

### Materials and methods

### Tumours and tumour growth rate

AT84 and 478 are both fairly well differentiated murine squamous cell carcinoma lines, derived from spontaneous tumours of the oral and vaginal mucosa respectively. An early, more slowly growing (AT478/4) and a late, much faster (AT478/25) generation of one tumour line were compared.

One $mm^3$ fragments of donor tumour were transplanted subcutaneously under pentobarbital anaesthesia into the flank of syngenic Neuherberg C3H mice using a trochar. Tumour growth was followed by caliper measurements and volume doubling times were estimated by fitting a Gompertz growth curve to the measured data.

*Labelling with DNA precursors*

For *in vivo* labelling, i.p. injections of $2\,mCi\,kg^{-1}$ $^3$H-TdR (H-5-methyl Thymidine, specific activity $185\,MBq\,mmol^{-1}$, Code No. TRA 61, Amersham Buchler, Braunschweig, Germany) or $50\,mg\,kg^{-1}$ BrdUrd (bromo-deoxyuridine, Sigma) diluted in saline to a volume of 0.1–0.2 ml were given per mouse.

For *in vitro* labelling, tumour slices of 0.3 mm were incubated with 0.4 $\mu$M $^3$H-TdR or 50 $\mu$M BrdUrd in Eagle medium at 37°C and at 2 atm $O_2$ pressure for 1 h. According to studies of Böswald *et al.*, 1990, this incubation period was chosen as equivalent to the *in vivo* availability time of tracers given by i.p. injection. The problem of reduced precursor uptake in the depth of tissue blocks (Denekamp & Kallman 1973; Chavaudra *et al.*, 1979) was successfully circumvented by incubating very thin tissue slices. In order to exclude toxic effects, BrdUrd concentrations of 50 $\mu$M and 1 $\mu$M were compared in one experiment.

*Detection of labelled cells*

*Flow cytometry (FCM)* In order to estimate $T_s$, BrdUrd was injected 1–4 h before tumour excision. Tumours were then excised, cut into several pieces and dropped into cold 70% ethanol. Fixed tumour pieces were stored at 4°C in the dark until staining. Tissue preparation and staining for flow cytometry has been described previously (Begg *et al.*, 1988). Briefly, the fixed tumour pieces were cut into smaller pieces and incubated in a pepsin solution to produce a suspension of nuclei. This was followed by partial acid denaturation of DNA, incubation with a mouse monoclonal antibody specific for DNA-incorporated BrdUrd, incubation with an FITC-conjugated anti-mouse antibody and addition of propidium iodide (PI) and RNase to stain total DNA. Flow cytometry was carried out using a FACStar flow cytometer (Becton Dickinson, Belgium) using excitation at 488 nm and collecting the green (FITC; BrdUrd) and red (PI; DNA) fluorescence signals using the following filters: 515–545 nm band pass (green), 650 nm long pass (red).

*Immunohistochemistry (IHC)* Two to four neighbouring, equatorial slices of 0.8 mm thickness were fixed in 2% paraformaldehyde in phosphate buffer, washed overnight in 6.8% sucrose in phosphate buffer and then embedded into glycolmethacrylate (Technovit 8100) as previously described (Schultz-Hector & Haghayegh, 1993). Three $\mu$m tissue sections were mounted on poly-L-lysine coated slides. Labelled nuclei were visualised by $^3$H-TdR autoradiography, BrdUrd-immunohistochemistry, or by combination of both. For double staining, protease treatment with 0.1% trypsin in 0.1% $CaCl_2$ at ph 7.8 for 10 min at 37°C, followed by 0.4% pepsin in 0.01 M HCl for 60 min at 37°C was performed before autoradiography. Slides were then coated with Kodak NTB 2 nuclear track emulsion diluted at 1:3 with distilled water. After exposure for 10 days at 4°C in the dark, autoradiographs were developed. In order to make the autoradiographic coating permeable for antibodies, slides were then dipped for 25 s into 0.2% trypsin in PBS at ph 7.2. Following DNA denaturation by 2 M HCl for 40 min, sections were incubated for 20 min with normal goat serum 1:20, for 2 h with mouse-anti-BrdUrd 1:40 (37°C), for 30 min with goat-anti-mouse 1:20 and finally for 30 min with alkaline-phosphatase-anti-alkaline-phosphatase (APAAP) complex. The alkaline phosphatase reaction was carried out using a naphthol AS-Bi-phosphate as substrate and new fuchsin as chromogen. All antibodies were obtained from DAKO (DAKO, Hamburg, Germany) and were diluted in 1% bovine serum albumin solution in PBS. Nuclei were counterstained with haematoxilin and sections coverslipped with gelatin. If not specified otherwise, reactions were carried out at room temperature.

Pilot experiments indicated that the double staining procedure as described did not modify the labelling index of either BrdUR or $^3$H-TdR. However, the conditions (time, concentration, temperature) of trypsin treatment after development of autoradiographies were very critical.

*Estimation of LI and $T_s$*

*Flow cytometry* The labelling index was obtained from cell numbers found in two windows placed around the FITC-labelled cells and total cells in cytogrammes of green *vs* red fluorescence (Begg *et al.*, 1985). The distinction between labelled and unlabelled cells was clear cut in these experimental tumours, with small differences in window placements having little or no influence on the estimated LI. At times longer than $TG_2$, two separate labelled subpopulations were visible, representing undivided and divided cells. In this case, the number of divided cells (at the $G_1$ position) were divided by two when calculating the LI (Begg *et al.*, 1985). Since it has been shown that unlabelled $G_2$ cells have very little effect on the overall LI, they were not taken into account (Begg *et al.*, 1989). Both the tumours were aneuploid with DNA indices of approximately 1.7. Few labelled cells were seen above the diploid peak. The windows were placed around the aneuploid tumour population only. $T_s$ was calculated by first estimating the parameter Relative Movement (RM) using a window placed around the undivided labelled cells and measuring their position relative to $G_1$ cells (Begg *et al.*, 1985). $T_s$ was then calculated according to the equation:

$$T_s = 0.5 \times t/(RM - 0.5),$$

where t is the time between BrdUrd injection and tumour excision. Again, only the aneuploid population was included.

*Histology* The transit of cells through S-phase was followed by sequential application of $^3$H-TdR and BrdUrd at intervals of 1–8 h. Tumours were excised and fixed 1 h after application of the second label. In histological sections the proportions of unlabelled, BrdUrd-labelled, $^3$H-TdR-labelled and double labelled tumour cell nuclei were determined. Tumour cell nuclei were relatively large and pale and in most cases could readily be distinguished from endothelial cells, fibroblasts or other host cells. For each labelling interval three tumours were studied, from each tumour a total of 6000–17,500 nuclei were counted from 2–4 sections. The labelling index (LI) was determined as percentage of BrdUrd positive nuclei of all counted nuclei.

With increasing time interval between injections, the proportion of nuclei labelled with BrdUrd but without $^3$H-TdR *vs* all BrdUrd labelled nuclei increases (Figure 4). If we denote this proportion with p, the time interval between the two labels with t, and with $T_s$ that point in time when p reaches 1 (100%), this can be expressed as

$$p = \begin{cases} 0 & t \leqslant 0 \\ \beta \cdot t \quad \text{for} & 0 < t < T_s \\ 1 & t \geqslant T_s, \end{cases}$$

This implies that for values of t between 0 and $T_s$, the equation relating p and t is a straight line starting at the origin. For values $\geqslant T_s$, p equals 1 (100%). From the fact that two sections of the relation must meet at $t = T_s$, one can derive the continuity condition

$$T_s = 1/\beta.$$

The parameters $\beta$ and $T_s$ and their standard errors were determined by nonlinear regression techniques (Seber & Wild, 1989). The computer programme used was BMDPAR (Dixon *et al.*, 1990). The aim of the experiment was rather to compare different groups of tumours within this study than to establish absolute values. Therefore no attempt was made to correct for the nonlinear age distribution of proliferating cells. Since for tumours of quite different growth and cell kinetic characteristics very similar values of $\lambda$ were calculated, there is no reason to assume that the age distribution of the tumours in the present study should be different from each other (Wilson *et al.*, 1992).

### Results

### Labelling index

In AT84 as well as in AT478/25 tumours of 100 mg size, comparison of *in vitro* or *in vivo* application of BrdUrd and detection by FCM of IHC, did not reveal any significant or systematic differences in LI. (Table I). No consistent trend was seen between *in vitro vs in vivo* LI, or between histology *vs* FCM LI. The overall average LIs were 22.8% and 22.1% in AT84 and AT478/25 respectively.

### S-phase duration

When DNA precursors were given *in vivo*, the $T_s$ values derived from histological evaluation and from flow cytometry were similar (Table I). However, when double labelling with $^3$H-TdR and BrdUrd was attempted *in vitro*, the second label was not incorporated when intervals exceeded 1 h. Similarly, there was no relative movement of labelled cells in tumours which were incubated in BrdUrd-free medium under hyperbaric oxygen conditions for 1–4 h after *in vitro* labelling with either 50 μM or 1 μM BrdUrd (Figure 1). For this reason, only *in vivo* $T_s$ estimates are included in Table I. The estimates of $T_s$ *in vivo* from FCM (Relative Movement method) and IHC (double labelling method) were in good agreement.

In order to investigate the reason for this failure of cells to proceed through the cell cycle *in vitro*, tumour slices were re-incubated in medium at 37°C under hyperbaric oxygen conditions for time periods up to 6 h, before labelling with 50 μM BrdUrd. The LI determined histologically decreased continuously after pre-incubation times of more than 3 h (Figure 2a). In contrast, the LI determined by FCM was unchanged throughout the experiment (Figure 2b). However, the fluorescence intensity of labelled nuclei, a measure of the DNA synthesis rate, decreased dramatically with increasing pre-incubation periods (Figure 2c). This indicates a much higher sensitivity of BrdUrd detection by FCM as compared to IHC. When growth factors were added to the medium during the pre-incubation period in the form of 10% foetal calf serum, a similar decrease in DNA synthesis rate was observed.

### Influence of tumour size and growth rate on $T_{pot}$

Volume doubling times Td as well as potential doubling times $T_{pot}$ of AT84/4 and AT478/25 were very similar in 100 mg tumours (Table II). With increasing tumour size (600 mg) however, there was a more pronounced reduction in tumour growth in AT84 as compared to AT478/25. The early, more slowly growing generation of AT478 showed much longer Td and $T_{pot}$ in small tumours but only a very moderate increase in both parameters with tumour size. The cell loss factor was greater in this tumour than in the two fast growing tumours.

A separate analysis of the two components of $T_{pot}$, LI and $T_s$, revealed that the size related increase in $T_{pot}$ in AT84 and AT478/25 was due to both a reduction of LI and a significant protraction of $T_s$. Comparison of the fast and the slowly growing passage of AT478 shows, that the $T_s$ in the two tumour generations was comparable while the LI of AT478/4 was very low at 100 mg and fully accounts for the observed prolongation in $T_{pot}$.

### Intratumour variability of $T_s$

All three tumours investigated roughly displayed a similar spatial pattern of proliferation. The tumour periphery usually showed a high density of labelled nuclei, bordering on a zone of viable, but less actively proliferating tumour tissue which was surrounding central areas of necrosis. Necrotic areas were minimal in small, i.e. 100 mg tumours and quite extensive in large, i.e. 600 mg tumours. Although this arrangement was predominant, there were occasionally necrotic areas close to the tumour capsule or chords of viable tumour tissue extending into necrotic areas. Within viable areas three different labelling patterns could be distinguished and could often be observed in one and the same tumour section: There

**Figure 1** Relative Movement as a function of time interval after *in vivo* (circles) or *in vitro* (triangles) labelling of AT84 (**a**) and AT478/25 (**b**). Plotted are mean values of three tumours per interval ± s.d.

**Table I** Comparison of different techniques of labelling and detecting cells in S-phase. Values of LI represent means ± s.e.m., $n = 9$; $T_s$ estimates were obtained as described; deviations are given as s.e.m.

| | *AT84* | *AT84* | *AT478/25* | *AT478/25* |
|---|---|---|---|---|
| | FCM | histology | FCM | histology |
| LI *in vivo* (%) | 27.8 ± 7.2 | 22.2 ± 0.9 | 19.1 ± 1.4 | 23.9 ± 0.8 |
| LI *in vitro* (%) | 22.3 ± 1.0 | 19.3 ± 0.5 | 21.4 ± 1.1 | 24.1 ± 0.8 |
| $T_s$ *in vivo* (h) | 10.95 ± 0.57 | 10.9 ± 0.8 | 9.86 ± 0.89 | 10.1 ± 0.4 |

were areas where proliferating cells appeared to be randomly distributed (Figure 3a). Often, tumour cells were arranged in cell nests, which were surround by blood vessels and which showed actively proliferating cells in their outer cell layers (Figure 3b). On the other hand, chords of tumour cells surrounding a supporting blood vessel with maximum proliferative activity in their centre could also be found (Figure 3c). In the more slowly growing AT478/4, the two latter patterns were predominant, while in fast growing tumours AT478/25 and AT84, and especially in the tumour periphery of small tumours, a random staining pattern prevailed.

**Figure 2** Effect of pre-incubation of tumour slices in medium at 37°C and under hyperbaric oxygen conditions for time periods up to 6 h on the BrdUrd labelling index. Each symbol represents the mean value ± s.d. of three tumours. **a**, Histological BrdUrd LI as a function of the length of time of pre-incubation. **b**, FCM BrdUrd LI as a function of the length of time of pre-incubation. **c**, Fluorescence intensity of BrdUrd positive nuclei in FCM as a function of the length of time of pre-incubation.

**Figure 3** Photomicrograph of murine scc's, after *in vivo* double labelling with $^3$H-TdR and BrdUrd at 1 h interval. $^3$H-TdR is detected by autoradiography (black silver grains), while BrdUrd is visualised by APAAP immunohistochemistry (bright red nuclear staining). **a**, Peripheral area of At84, showing a random distribution of labelled nuclei. Most labelled nuclei are positive for both markers. Only occasional nuclei are positive for only $^3$H-TdR (arrow) or BrdUrd (double arrow). The line represents 50 μm. **b**, Marginally labelled tumour cell nest of AT478/25. The line represents 100 μm. **c**, Labelled tumour cells surrounding a blood vessel, AT478/25. The line represents 100 μm.

**Table II** Cell kinetic and tumour growth parameters for small and large tumours of each tumour line

| | *AT84/4* | | *AT478/25* | | *AT478/4* | |
|---|---|---|---|---|---|---|
| | 100 mg | 600 mg | 100 mg | 600 mg | 100 mg | 600 mg |
| Td (95% c.l.) (d) | 2.6 (2.2–3.2) | 15.1 (10–30) | 2.7 (2.3–3.1) | 11 (7.7–17) | 6.4 (5.9–7.1) | 19.5 (14–33) |
| $T_{pot}$ (d) | 2.0 | 5.2 | 1.8 | 3.5 | 3.6 | 4.7 |
| LIs ± s.e.m. (%) | 22.2 ± 0.9 | 13.9 ± 0.4 | 23.9 ± 0.9 | 14.6 ± 0.9 | 11.7 ± 0.8 | 11.23 ± 0.7 |
| $T_s$ ± s.e.m. (h) | 10.8 ± 0.8 | 17.4 ± 0.9 | 10.1 ± 0.4 | 12.4 ± 2.4 | 10.0 ± 0.2 | 12.6 ± 0.6 |
| cell loss (%) | 23 | 65.6 | 33.3 | 67.3 | 43.8 | 75.9 |

In AT478/25 tumours, the actively proliferating tumour periphery was analysed separately from the more quiescent, but still viable central areas in order to quantitate regional differences in $T_{pot}$. In the periphery, the labelling index was 35% and a $T_s$ of 10 h was estimated, while in the centre the LI dropped to 10.8% and $T_s$ increased to 22.5 h. Thus, within one tumour, different local values of values ranging from 1 to 7 days can be calculated (Figure 4). In terms of three-dimensional tissue volumes however, the quiescent tumour centre (Figure 4b) represents only a very minor proportion of the tumour volume. Since the LI is low in the tumour centre, this cell population is relatively under-represented in an overall analysis of tumour $T_s$ (Table I), taking only labelled nuclei into account.

### Discussion

This study provides several comparisons of techniques to measure cell proliferation kinetics in tumours. These comparisons will be discussed in turn, followed by some conclusions and recommendations.

### *Flow cytometry* vs *immunohistochemistry*

LI and $T_s$ values obtained on the same tumours by FCM and IHC were in good agreement. The tumours studied were both aneuploid so that flow cytometric distinction between tumour and host cells could be made based on DNA content. The DNA index of both tumours was approximately 1.7, so that an overlap was possible between normal $G_2$ cells and tumour $G_1$ and S-phase cells. Few if any labelled cells were seen in the unequivocally normal cell population, however, so that any inaccuracies in LI due to population overlap are likely to be small. This was not the case in the clinical studies of Bennet and colleagues (1992), or in the human xenograft studies of Parkins *et al.* (1991), where unlabelled stromal cells could not readily be distinguished from diploid tumour cells, leading to an underestimate of LI by FCM. Aneuploid human tumour cells labelled *in vitro* with BrdUrd were also found to have a higher LI in the study of Gasinska *et al.* (1989), although a good correlation was found for LI between the FCM and IHC methods when all cells were included with both methods. These data suggest that there is little difference in the ability to discriminate between labelled and unlabelled cells by the two techniques, although LI for diploid tumours may be underestimated by FCM, and possibly for IHC if a morphological distinction between normal and malignant cells is difficult.

**Figure 4** Separate analysis of $T_s$ in the periphery (**a**) and centre (**b**) of 600 mg AT478/25. With increasing time interval between application of $^3$H-TdR and BrdUrd, the proportion of nuclei labelled for BrdUrd only in relationship to all labelled nuclei increases. $T_s$ estimates were $10.3 \pm 0.26$ h in the periphery and 22.5 in the centre, while corresponding LI values were $35.1 \pm 4.1$% and $10.8 \pm 1.7$% respectively. Symbols represent single tumours.

### *$^3$H-TdR* vs *BrdUrd*

Comparison of LIs obtained after labelling with $^3$H-TdR or BrdUrd has yielded a good correlation in some studies (Meyer *et al.*, 1989; Wilson *et al.*, 1985), while other authors reported a greater sensitivity of the BrdUrd technique (Knapp, 1992). In the present study, no difference between $^3$H-TdR and BrdUrd LI values were seen, indicating the equivalence of the two techniques in our hands. Simultaneous application of $^3$H-TdR and BrdUrd *in vivo*, however, was reported to require an increase in either $^3$H-TdR dose or autoradiographic exposure time in order to obtain a constant $^3$H-TdR LI (Hume & Thompson, 1989), while the BrdUrd LI was the same in single and double labelling experiments. In order to prevent a possible inhibition of $^3$H-TdR uptake by BrdUrd, $^3$H-TdR was always given prior to BrdUrd in the present study. Since there was no systematic or significant difference in $^3$H-TdR and BrdUrd LIs in double labelling experiments, an interaction between these two precursors appears to have been insignificant.

### *Can $T_s$ of explants be measured* in vitro?

One goal of the study was to assess the feasibility of estimating $T_s$ after *in vitro* labelling of tumour biopsies, thus circumventing the necessity of systemic application of DNA precursors in patients. Although LI values were not altered by pre-incubation of tumour slices for up to 3 h at 37°C and much longer time periods at lower temperatures, neither double labelling nor progression through the cycle measured by flow cytometry could be achieved. This could not be modified by hyperbaric oxygen, reduction of precursor concentrations to non-toxic levels or the addition of extra growth factors in the form of foetal calf serum. Similar difficulties had been encountered using radioactive labelling techniques, as summarised by Steel (1977). Tumour cells with a DNA content between $G_1$ and $G_2$ but negative for BrdUrd have been described in several studies (de Fazio *et al.*, 1987; Wilson *et al.*, 1985). In addition, S-phase arrest can be induced in leukaemia cells by incubation with ARA-C, IFN or an IL-1 receptor antagonist (Preisler *et al.*, 1992). However, S-phase arrest is not a usual physiological mechanism of cell cycle control but probably occurred in these experiments because of the *in vitro* conditions. It is not clear how this can be circumvented, although other oxygenation conditions, incubation times and/or growth factors need to be tested. We conclude that a purely *in vitro* estimation of $T_s$ by relative movement or double labelling is impossible using standard techniques.

### *Is measurement methods: single* vs *double labelling*

The double labelling technique used in the present study for histological assessment of $T_s$ combines $^3$H-TdR autoradiography with BrdUrd IHC, as suggested by Hamada (1985). It is based on quantifying the proportion of cells exiting or entering S-phase during the interval between labels and thus containing only one label rather than two (Wimber & Quastler, 1963). Double labelling can also be performed using two different radioisotopes to label thymidine, which can then be deteced in different layers of autographic emulsion (Schultze

*et al.*, 1976). Alternatively, non-radioactive techniques have been developed using two different thymidine analogues which can be recognised by specific antibodies. However, cross-reactivity of these antibodies could be a problem in both IHC methods (Shibui *et al.*, 1989; Hoshino *et al.*, 1986) and FCM (Bakker *et al.*, 1989; Raza, 1987). Furthermore, histological distinction between bound antibodies, i.e. the distinction of unlabelled, single or double labelled nuclei is entirely based on a visual distinction of colours, which could be problematic, e.g. in nuclei labelled strongly with one label and weakly with the other. These difficulties can be avoided by combining $^3$H-TdR autoradiography with BrdUrd IHC. The risk of confusing the two labels or of mistaking a double labelled for a singly labelled nucleus is minimal when one precursor is recognised by red staining in the section plane and the other by silver grains in the photographic emulsion above the section (Figure 3a). $T_s$ estimates have also been derived from quantitative analysis of nuclear uptake of one single radioactive DNA precursor (Dörmer, 1973). However, this requires analysis of the whole nucleus, which is only possible in cell smears.

In this study, the $T_s$ values measured by FCM, using a single analog and the Relative Movement method, were insignificantly different from those measured by the double label IHC/autoradiography technique (Table I). The present technique using a combination of IHC and autoradiography could also be applied to clinical material. *In vivo* labelling with BrdUrd could be followed by *in vitro* labelling with $^3$H-TdR, provided that tumour exision is delayed for several hours, until a time interval suitable for measurement of cell cycle progression has elapsed and *in vitro* incubation can be performed immediately after surgery. In addition to the advantages mentioned above, this would mean that administration to the patient of two labels could be avoided. However, neither this or other studies have yet shown that double labelling has advantages over the FCM single labelling method.

### *Tumour size dependence*

Comparison of small, i.e. 100 mg and large, i.e. 600 mg tumours showed a significant increase in $T_s$ with tumour size. While small tumours had little of no necrosis, large tumours had developed fairly extensive areas of necrosis which were presumably surrounded by a zone of hypoxic but viable cells. Since a retarding effect of hypoxia on cycle progression has been observed both *in vitro* (Born *et al.*, 1976) and *in vivo* (Shrieve & Begg, 1985), the tumour size related increase in $T_s$ may be due to increased tumour hypoxia. When tested functionally however, the time course of repopulation was found identical in small and large AT478 tumours (Kummermehr, 1993).

The LI and $T_s$ values obtained in the present study are longer than in most other reports on experimental tumours (reviewed by Denekamp, 1970 and by Steele, 1977; Carlton *et al.*, 1991). However, both tumour lines investigated in the present study were fairly well differentiated and slowly growing. Although the LI values reported here are higher than in most human tumours (Meyer & He, 1993), they are lower than in the majority of experimental tumours described (Denekamp, 1970 and Steele, 1968). A fast, late passage and a slow, early generation of one tumour line differed in LI, growth fraction and cell loss factor, but not in $T_s$.

### *Regional variations in LI and $T_s$*

In human head and neck squamous cell carcinomas, Bennett *et al.* (1992) described histological patterns of proliferation of tumour cell nests as either marginal, intermediate or random, with considerable local variation of LI. The present study shows that not only the LI but also the $T_s$ is subject to considerable regional variation within one tumour, ranging from 10–22.5 h. The values of $T_s$, LI and $T_{pot}$ thus represent overall means which are composed of a wide range of local values. However, the scale at which these extremes are being observed is rather small and should be below an average sized clinical tumour biopsy. This is in good agreement with the finding of a moderate to low intratumoural variability when several biopsies from one human tumour were compared (Begg *et al.*, 1988; Wilson *et al.*, 1988; Bennett *et al.*, 1992). Begg *et al.* (1988) reported that the variability in LI was considerably greater than that for $T_s$ in multiple biopsies taken from human tumours. This was not the case in our comparison of tumour periphery and centre, where both parameters changed by a factor of about two within a few millimeters. It therefore appears likely that the spatial resolution of comparing whole biopsies is not sufficient to detect regional variations in $T_s$.

Assuming that variations in $T_{pot}$ comparable to those observed in murine squamous cell carcinomas occur in human tumours, the important question would be which of these regional $T_{pot}$ values is the clinically relevant predictor of tumour repopulation during radiotherapy? The shortest values may approximate the maximum proliferative capacity of a tumour, while the longer values may be associated with hypoxia. Systematic performance of histological studies in conjunction with FCM, estimating the regional minimum and maximum $T_{pot}$ in pre-treatment biopsies of human tumours could answer this question and could possibly increase the predictive power of overall $T_{pot}$ measurements by FCM.

In summary, it appears in general that FCM and IHC methods give similar results for both LI and $T_s$. For laboratories without access to flow cytometers, IHC techniques are therefore clearly a good alternative for cell kinetic studies. FCM has the advantage of speed, however, and would be preferred if both systems are available. The big advantage of the IHC remains that morphological and positional information is retained. This could be a crucial advantage in diploid tumours for example, unlike those studied here, where no distinction can be made between tumour and normal cells on the basis of DNA content. FCM is only adequate in these situations if separate tumour markers can be measured simultaneously (Begg & Hofland, 1991; Raemakers *et al.*, 1986). Intratumoural variability can also better be studied with IHC techniques where only a gross estimate can be made with FCM. Finally, the present results suggest that $T_s$ cannot be accurately measured in fresh tumour explants by *in vitro* labelling, either by FCM or IHC methods. Therefore for patient studies requiring $T_s$ estimates, *in vivo* labelling remains the only choice at present.

### References

BAKKER, P.J.M., ATEN, J.A., TUKKER, C.J., BAREBDSEN, G.W. & VEENHOF, C.H.N. (1989). Flow cytometric analysis of experimental parameters for the immunofluorescent labeling of BrdUrdrd in various tumour cell lines. *Histochemistry*, **91**, 425–429.

BEGG, A.C., HOFLAND, I., VAN GLABBEKE, M., BARTELINK, H. & HORIOT, J.C. (1992). Predictive value of potential doubling time for radiotherapy of head and neck tumor patients: results from the EORTC Cooperative Group trial 22851. *Semin. Rad. Oncol.*, **2**, 22–25.

BEGG, A.C. (1993). Critical appraisal of *in situ* cell kinetic measurements as response predictors in human tumors. *Semin. Radiat. Oncol.*, **3**, 144–151.

BEGG, A.C., MCNALLY, N.J., SHRIEVE, D.C. & KÄRCHER, H. (1985). A method to measure the duration of DNA synthesis and the potential doubling time from a single sample. *Cytometry*, **6**, 620–626.

BEGG, A.C. (1989). Derivation of cell kinetic parameters from human tumours after BUdR or IUdR labelling. In *Scientific Basis of Modern Radiotherapy*, BIR Report 19: 113–119.

BEGG, A.C., MOONEN, L., HOFLAND, I., DESSING, M. & BARTELINK, H. (1988). Human tumour cell kinetics using a monoclonal antibody against iododeoxyuridine: Intratumour sampling variations. *Radiother. Oncol.*, **11**, 337–347.

BEGG, A.C. & HOFLAND, I. (1991). Cell kinetic analysis of mixed populations using three color fluorescence flow cytometry. *Cytometry*, **12**, 445–454.

BENNETT, M.H., WILSON, G.D., DISCHE, S., SAUNDERS, M.I., MARTINDALE, C.A., ROBINSON, B.M., O'HALLORAN, A.E., LESLIE, M.D. & LAING, J.H.E. (1992). Tumour proliferation assessed by combined histological and flow cytometric analysis: implications for therapy in squamous cell carcinoma in the head neck. *Br. J. Cancer*, **65**, 870–878.

BORN, R., HUG, O. & TROTT, K.-R. (1976). The effect of prolonged hypoxia on growth and viability of chinese hamster cells. *Int. J. Radiat. Oncol. Biol. Phys.*, **1**, 687–697.

BÖSWALD, M., HARASIM, S. & MAURER-SCHULTZE, B. (1990). Tracer dose and availability time of thymidine and bromodeoxyuridine: application of bromodeoxyuridine in cell kinetic studies. *Cell Tissue Kinet.*, **23**, 169–181.

CARLTON, J.C., TERRY, N.H.A. & WHITE, R.A. (1991). Measuring potential doubling times of murine tumors using flow cytometry. *Cytometry*, **12**, 645.

CHAVAUDRA, N., RICHARD, J.M. & MALAISE, E.P. (1979). Labelling index of human squamous cell carcinomas. Comparison of *in vivo* and *in vitro* labelling methods. *Cell Tissue Kinet.*, **12**, 145–152.

DEFAZIO, A., LEARY, J.A., HEDLEY, D.W. & TATTERSALL, H.N. (1987). Immunohistochemical detection of proliferating cells *in vivo*. *J. Histochem. Cytochem.*, **35**, 571–577.

DENEKAMP, J. (1970). The proliferation kinetics of animal tumors. *Cancer Res.*, **30**, 393–400.

DENEKAMP, J. & KALLMAN, R.F. (1973). *In vitro* and *in vivo* labelling of animal tumours with tritiated thymidine. *Cell Tissue Kinat.*, **6**, 217–227.

DIXON, W.J., BROWN, M.B., ENGELMANN, L. & JENNRICH, R.I. (1990). BMDP statistical software manual. Volume 1. University of California Press, Berkeley.

DÖRMER, P. (1973). Kinetics of erythropoietic cell proliferation in normal and anemic men. A new approach using quantitative 14C autoradiography. *Prog. Histochem. Cytochem.*, **6**, 1–83.

GASINSKA, A., WILSON, G.D. & URBANSKI, K. (1989). Labelling index of gynecological tumours assessed by bromodeoxyuridine staining *in vitro* using flow cytometry and histochemistry. *Int. J. Radiat. Biol.*, **56**, 793–796.

HAMADA, S. (1985). A double labelling technique combining $^{3}$H-thymidine autoradiography with BrdUrd immunocytochemistry. *Acta Histochem. Cytochem.*, **18**, 267–270.

HOSHINO, T., NAGASHIMA, T., CHO, K.G., MUROVIC, J.A., HODES, J.E., WILSON, C.B., EDWARDS, M.S.B. & PITT, L.H. (1986). S-phase fraction of human brain tumors *in situ* measured by uptake of bromodeoxyuridine. *Int. J. Cancer*, **38**, 369–374.

HUME, W.J. & THOMPSON, J. (1989). Double labelling of tissue combining tritiated thymidine autoradiography with immmunodetection of bromodeoxyuridine: the autoradiographic significance of inhibition of thymidine incorporation into DNA by bromodeoxyuridine given simultaneously. *Cell Tissue Kinet.*, **22**, 393–399.

KNAPP, P.E. (1992). The cell cycle of glial cells grown *in vitro*: an immunocytochemical method of analysis. *J. Histochem. Cytochem.*, **40**, 1405–1411.

KUMMERMEHR, J. (1993). The time factor in experimental tumors. *Proceedings of the 4th International Conference on Time, Dose and Fractionation in Radiation Oncology*. Fowler, J. (ed.). (in press).

MEYER, J.S., PREY, M.U., BABCOCK, D.S. & MCDIVITT, R.W. (1986). Breast carcinoma cell kinetics, morphology, stage, and host characteristics. A thymidine labelling study. *Lab. Invest.*, **54**, 41–51.

KNAPP, P.E. (1992). The cell cycle of glial cells grown *in vitro*: an immunocytochemical method of analysis. *J. Histochem. Cytochem.*, **40**, 1405–1411.

KUMMERMEHR, J. (1993). The time factor in experimental tumors. *Proceedings of the 4th International Conference on Time, Dose and Fractionation in Radiation Oncology*. Fowler, J. (ed.). (in press).

MEYER, J.S., PREY, M.U., BABCOCK, D.S. & MCDIVITT, R.W. (1986). Breast carcinoma cell kinetics, morphology, stage, and host characteristics. A thymidine labelling study. *Lab. Invest.*, **54**, 41–51.

MEYER, J.S., NAUERT, J., KOEHM, S. & HUGHES, J. (1989). Cell kinetic studies of human tumors by *in vitro* bromodeoxyuridine labeling. *J. Histochem. Cytochem.*, **37**, 1449–1454.

MEYER, J.S. & HE, W. (1993). Cell proliferation measurements by bromodeoxyuridine or thymidine incorporation: clinical correlates. *Semin. Radiat. Oncol.*, **3**, 126–134.

PREISLER, H.D., GOPAL, V., BANAVALI, S.D., FINKE, D. & BOKARI, S.A.J. (1992). Multiparameter assessment of the cell cycle effects of bioactive and cytotoxic agents. *Cancer Res.*, **52**, 4090–4095.

PARKINS, C.S., BUSH, C., PRICE, P. & STEEL, G.G. (1991). Cell proliferation in human tumour xenografts: measurement using antibody labelling against bromodeoxyuridine and Ki-67. *Cell Prolif.*, **24**, 171–179.

RAMAEKERS, F.C., BECK, H.L., FEITZ, W.F., OUD, P.S., DEBRUYNE, F.M., VOOIJS, G.P. & HERMAN, C.J. (1986). *Anal. Quant. Cytol. Histol.*, **8**, 271–280.

RAZA, A., MAHESHWARI, H., YASIN, Z., MANDAVA, N., MAYERS, G. & PREISLER, H.D. (1987). A new method for studying cell cycle characteristics in ANLL using double-labeling with BrdUrd and $^{3}$H-Tdr. *Leukemia Res.*, **11**, 1079–1085.

SHRIEVE, D.C. & BEGG, A.C. (1985). Cell cycle kinetics of aerated, hypoxic and re-areated cells *in vitro* using flow cytometric determination of BrdUrd incorporations. *Cell & Tissue Kinet.*, **18**, 641–651.

SCHULTZE, B., MAURER, W. & HAGENBUSCH, H. (1976). A two emulsion autoradiographic technique and the discrimination of the three different types of labelling after double labelling with $^{3}$H-and $^{14}$C-thymidine. *Cell Tissue Kinet.*, **9**, 245–255.

SCHULTZ-HECTOR, S. & HAGHAYEGH, S. (1993). b-FGF expression in human and murine squamous cell carcinomas (scc) and its relationship to regional endothelial cell proliferation. *Cancer Res.*, **53**, 1444–1449.

SEBER, G.A. & WILD, C.J. (1989). *Nonlinear regression*. Chapter 9. Wiley, New York.

SHIBUI, S., HOSHINO, T., VANDERLAAN, M. & GRAY, J.W. (1989). Double labeling with iodo- and bromodeoxyuridine for cell kinetic studies. *J. Histochem. Cytochem.*, **37**, 1007–1011.

SILVESTRINI, R., MOLINARY, R., COSTA, A., VOLTERRANI, F. & GARGANI, G. (1984). Short-term variation in labelling index as a predictor of radiotherapy response in human oral cavity carcinoma. *Int. J. Radiat. Oncol. Biol. Phys.*, **10**, 965–970.

STEEL, G.G. (1977). *Growth kinetics of tumours*. Clarendon Press: Oxford. pp. 106–111.

TUBIANA, M., PEJOVIC, M.H., KOSCIELNY, S., CHAVAUDRA, N. & MALAISE, E. (1989). Growth rate, kinetics of tumor cell proliferation and long-term outcome in human breast cancer. *Int. J. Cancer*, **44**, 17–22.

WHITE, R.A., TERRY, N.A.H., BEGGERLY, K.A. & MEISTRICH, M.L. (1991). Measuring cell proliferation by relative movement. I. Introduction and *in vitro* studies. *Cell Prolif.*, **24**, 257–270.

WILSON, G.D., MCNALLY, N.J., DUNPHY, E., KÄRCHER, H. & PFRAGNER, R. (1985). The labelling index of human and mouse tumours assessed by bromodeoxyuridine staining *in vitro* and *in vivo* and flow cytometry. *Cytometry*, **6**, 641–647.

WILSON, G.D., MCNALLY, N.J., DISCHE, S., SAUNDERS, M.I., DES ROCHERS, C., LEWIS, A.A. & BENNETT, M.H. (1988). Measurement of cell kinetics in human tumours *in vivo* using bromodeoxyuridine incorporation and flow cytometry. *Br. J. Cancer*, **58**, 423–431.

WILSON, G.D., MARTINDALE, C.A., SORANSON, J.A., CARL, U.M. & MCNALLY, N.J. (1992). Proliferative changes in two murine tumours in response to single or fractionated doses of X-rays. *Cell Prolif.*, **25**, 415–430.

WIMBER, D.E. & QUASTLER, H. (1963). A $^{14}$C- and $^{3}$H-thymidine double labelling technique in the study of cell proliferation in tradescantia root tip. *Exp. Cell Res.*, **30**, 8–22.